# Left atrial morpho-functional remodeling in atrial fibrillation assessed by three dimensional speckle tracking echocardiography and its value in atrial fibrillation screening

**DOI:** 10.1186/s12947-022-00282-5

**Published:** 2022-05-03

**Authors:** Lilian Bao, Lei Cheng, Xiufang Gao, Fangying Yan, Huihua Fan, Ying Shan, Yong Li, Haiming Shi, Guoqian Huang, Liwen Bao

**Affiliations:** 1 Department of Cardiology, Zhongshan Hospital, Fudan University, Shanghai, China; 2 Department of Cardiology, Huashan Hospital, Fudan University, Shanghai, China

**Keywords:** Three dimensional speckle tracking echocardiography, Atrial fibrillation, Left atrial function, Left atrial strain, Remodeling

## Abstract

**Background:**

Three dimensional speckle tracking echocardiography (3D STE) is a novel technique combining 3D echocardiography and speckle tracking analysis. 3D STE software dedicated to the left atrium (LA) was recently available. Our study aimed to assess (1) atrial fibrillation (AF) related LA morpho-functional remodeling using 3D STE and (2) value of LA function parameters in identifying paroxysmal AF (PAF).

**Methods:**

One hundred thirty-nine PAF, 109 persistent AF (Per-AF) and 59 non-AF subjects underwent 3D STE. LA phasic volumes and total LA emptying fraction (LAEF) were obtained and used to calculate passive (pLAEF) and active LA emptying fraction (aLAEF) based on atrial contraction. LA longitudinal and circumferential strain representing reservoir (LASr/LASrc), conduit (LAScd/LAScdc) and pump (LASct/LASctc) function were also assessed.

**Results:**

3D STE was found to have good reproducibility. Increase of LA volumes and decrease of parameters representing LA reservoir and pump function were independently associated with AF as well as AF burden. The correlations between LA emptying fraction and LA circumferential strain representing the same function were always stronger than those with LA longitudinal strain (*p* < 0.001). Minimal LA volume, LAEF, aLAEF, LASrc and LASctc can be used to accurately differentiate PAF from non-AF subjects (AUC > 0.8) with great sensitivity and specificity.

**Conclusions:**

Assessing LA remodeling in AF using 3D STE was feasible. AF and AF burden were independently associated with LA enlargement and impairment of reservoir and pump function but not conduit function. LA function parameters can indicate underlying PAF and thus can guide AF screening strategy.

## Background

Atrial fibrillation (AF) is one of the most common arrhythmias, with an estimated prevalence between 2 and 4% worldwide [1]. The burden of the disease is rising due to aging of the population and increasing prevalence of cardiovascular disease [2]. However, the pathogenesis of AF hasn’t been fully clarified until now. Current studies suggested it may begin with hemodynamic overload and/or atrial remodeling mainly including left atrial (LA) enlargement and fibrosis which interferes with normal electric conduction and cause reentry circuits [3–5]. On the other hand, the presence of AF in turn attributes to LA dilation and function impairment [3].

The LA plays a phasic role during one whole cardiac circle, which importantly contributes to roughly 30% of the left ventricular (LV) stroke volume. During ventricular systole, the LA receives blood entering LA via pulmonary veins and thus serves as a “reservoir”. During early diastole, blood passively enters LV due to the pressure gradient between LA and LV, reflecting the “conduit” function of LA. Lastly, during late diastole, the LA acts as a booster pump, contracts actively and squeeze more blood into LV [6, 7]. To date, only LA maximal volume index is officially recommended in guidelines as an independent predictor for adverse cardiovascular outcomes [8], however, other LA function parameters have also been proven valuable and might even be more sensitive, including other LA volume parameters, LA emptying fraction and strain [9–12]. Strain, defined as the fractional change of length/width/thickness of one object in comparison to its original length/width/thickness, describes the deformation capacity of myocardium and thus reflect the mechanic function of heart chambers [13]. LA strain is a relatively new index to characterize LA function compared to other “conventional” echocardiographic measurements, however, some scholars think it might be superior to LA volumetric function parameters as it can distinguish LA active deformation from passive motion [14].

LA strain can be obtained by Tissue Doppler Imagine (TDI) or speckle tracking echocardiography (STE), with the latter being more widely used [14]. “Speckles” are acoustic markers of myocardium that move together with tissue during the cardiac circle. By tracking these speckles through the cardiac circle with certain software, we are able to analyse myocardial deformation function [15]. Three dimensional STE (3D STE) is a novel technique in the field of echocardiography, combining real-time 3D echocardiography with speckle tracking analysis. Theoretically, 3D STE overcomes several inherent limits of TDI and 2D STE such as angle dependency, “out of plane phenomenon”, geometric assumption, etc. [7, 16]. However, till now studies using this technique in LA function assessment is quite scarce and most current studies used softwares originally designed for LV instead of LA. Good news is that 3D STE software package dedicated to the LA was recently launched (4D LAQ, EchoPAC, GE Healthcare) which allows simultaneous analysis of LA volume and strain.

Therefore, in this study, we aimed to investigate if 3D STE can be used to comprehensively assess LA function and detect LA structural and functional changes in patients with AF. Besides, we also investigated the capacity of 3D STE derived LA parameters to identify PAF as well as their value in guiding AF screening strategy in clinical practice.

## Method

### Study design and population

This was a single center cross-sectional study approved by the Huashan Institutional Review Board. Patients with clearly diagnosed AF visiting Huashan Hospital from 2019.4.1 to 2020.1.15 and 2020.6.1 to 2021.1.10^1^ were continuously enrolled in this study after consent form assigned, meanwhile individuals without AF or major cardiovascular conditions other than a short history of mild hypertension visiting our hospital for health checkup were set as control group. Exclusion criteria included valvular AF, moderate to severe mitral/ tricuspid/ aortic stenosis or regurgitation, recent myocardial infarction within 3 months, acute pulmonary embolism, newly onset AF during or shortly after cardiac surgery, specific type of cardiomyopathy such as cardiac amyloidosis, obstructive hypertrophic cardiomyopathy, etc., decompensation stage of organ failure, and subjects unable to finish a successful echocardiography examination due to any reason.

### Classification of AF pattern

Patients with AF were divided into paroxysmal AF group (PAF) and persistent AF group (Per-AF) according to the 2020 ESC guideline by comprehensively considering their symptoms, 12 lead electrocardiogram (ECG), 24 h Holter or other data available. The Per-AF group actually included persistent AF, long standing persistent AF and permanent AF.

### Collection of clinical data

Subjects’ baseline clinical data including general health information and comorbidity were collected after enrolment mainly by reviewing their official medical records and comprehensive interviews with individual participants. The definitions for all clinical conditions were in accordance with the related guidelines [17–22].

### Conventional echocardiography examination

GE Vivid E95 System and a M5Sc-D transducer (frequency: 3.5-5 MHz) were used for the transthoracic echocardiography (TTE) examination, color doppler imaging, pulsed-wave Doppler echocardiography and tissue doppler imaging. Patients were positioned in left lateral decubitus position and connected to ECG monitor. All procedures were implemented by a sophisticated echocardiography technician with over 10 year experience using standard imaging plane following the 2019 and 2015 ASE guideline [8, 23]. The measurements we took included left ventricular end-diastolic diameter (LVEDD), left ventricular end-diastolic volume (LVEDV), left ventricular end-systolic volume (LVESV), left ventricular ejection fraction (LVEF) calculated by the Biplane Simpson method. Early (E) and late (A) filling velocity at the tip of mitral valve leaflet, E to A ratio, peak early (e’) and late (a’) diastolic mitral annular velocities at both septal and lateral sides were also obtained and E/e’ were calculated to evaluate LV filling pressure with e’ being the average of septal and lateral early diastolic velocities. LA diameter in 3 directions (suprainferior, mediolateral and anteroposterior) were measured at end systole just before the mitral valve opening. We also measured LA maximal volume (2DLAVmax) using Biplane Simpson method. LV diastolic function of each patient were comprehensively evaluated based on septal and lateral e’, E/e’, 2DLAVmax index to body surface area (2DLAVImax) as well as maximal tricuspid regurgitation velocity (TRMax) and graded as normal diastolic function, indeterminate diastolic function and diastolic dysfunction according to the 2016 EACVI guideline [24]. The above criteria was not applicable for patients with Per-AF, in which case E/(septal e’) ratio ≥ 11 was used to determine the existence of LV diastolic dysfunction. Effort was made to select matched RR intervals for E and e’ that were within 20% of the average heart rate.

### Speckle tracking echocardiography of LA

**Fig. 1:**
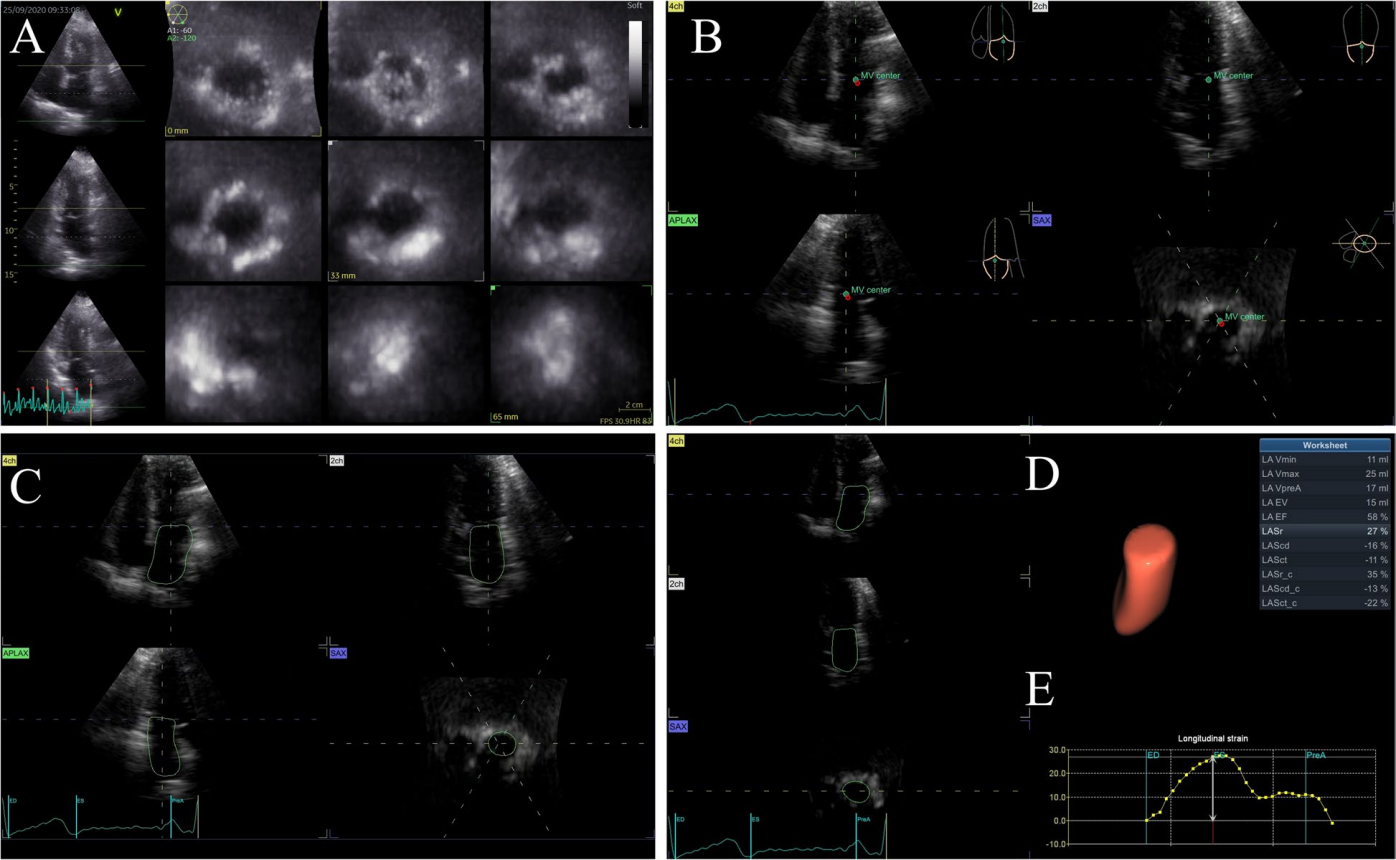
An example of motion tracking analysis of LA using 4D Auto LAQ, Echopac, GE Health. **A** Display of acquired LA 3D images showing apical four-chamber, two-chamber, three-chamber plane on the left and 9 slices of short-axis views from mitral annular plane to the atrial roof. **B** Landmark setting stage with three landmarks set at proper places according to the instruction. **C** Review stage with the LA endocardial contour traced. **D** A simulated model of reconstructed LA and output parameters. **E** LA strain/volume-time curve

**Fig. 2:**
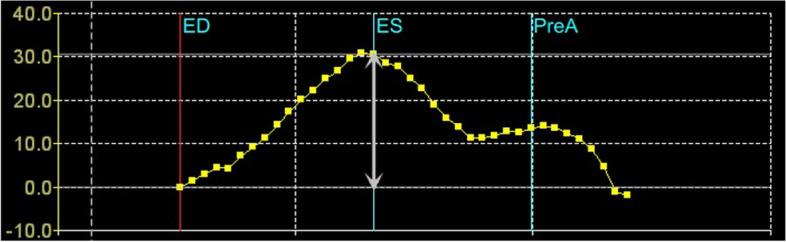
An example of the (longitudinal/circumferential) LA strain-time curve. ED = end ventricular diastole, ES = end ventricular systole, PreA = pre atrial contraction

3D speckle tracking echocardiography was performed right after the 2D echo exams using the same GE E95 system and a 4V-D (frequency: 1.5-4 MHz) transducer. Subjects were asked to maintain their position and breathe effortlessly. With good ECG monitor and clear visualization of the entire LA cavity at the apical 4-chamber view, 4D Large mode were switched into. Single-beat (if patients were under AF rhythm) or multi-beat (3 beats, if patients were under sinus rhythm) 3D LA datasets were acquired and stored with one breath hold at end expiration for optimal image reconstruction. It’s worth mentioning that all control and PAF subjects underwent acquisition under sinus rhythm while patients with Per-AF were under AF rhythm. Care was taken to avoid artifacts and volume rate was set at least above 12 volumes per second to satisfy speckle tracking between consecutive frames. Offline analysis was then performed using a commercially available semiautomatic software package (4D LAQ, EchoPAC, GE Healthcare). Three long-axis planes including apical 4-chamber, apical 2-chamber and apical 3-chamber together with 12 short-axis slices from mitral annular plane to LA roof were displayed on the screen (Fig. 1A). The user then needed to set 3 landmarks on the 3 long-axis planes by placing them at the centre of MV at the annular level in each plane (Fig. 1B). Then the endocardial border of LA as well as its movement through the entire cardiac cycle was traced and tracked by the system automatically and could be adjusted by the operator if necessary (Fig. 1C). After the motion-tracking analysis, the system output the following parameters: LA phasic volumes (maximal LA volume (LAVmax), minimal LA volume (LAVmin), LA volume at the onset of atrial contraction (LAVpreA) and LAVmax index to body surface area (LAVImax)), volumetric function parameters (total LA emptying volume (LAEV) and emptying fraction (LAEF)) and strain parameters including peak atrial longitudinal/circumferential strain (LASr/LASrc), early diastolic longitudinal/circumferential strain (LAScd/LAScdc), late diastolic longitudinal/circumferential strain (LASct/LASctc) along with an LA volume-time curve or LA strain-time curve (Fig. 1D&E). Note that the LA volume-time curve and LA strain-time curve followed the same shape. Another example of the (longitudinal/circumferential) LA strain-time curve was as presented in Fig. 2. The zero point refers to the end ventricular diastole. As the blood enters LA, the LA strain increases and achieves its maximal value at end ventricular systole on mitral valve opening. The peak strain value measures the reservoir function of LA and thus is also known as LA strain reservoir (LASr). LA serves as a booster pump and contracts actively during late ventricular diastole. A plateau can be seen on the strain-time curve, which corresponds to the beginning of LA contraction. The plateau value reflects the contraction function of LA, and thus is known as LA strain contraction (LASct). During early ventricular diastole, LA serves as a conduit, allowing blood passively flows into LV. LA strain that measures the LA conduit function, namely LAScd, can be calculated as LASct minus LASr. LASrc, LAScdc and LASctc hold the same meaning as LASr, LAScd and LASct, respectively, but describe LA deformation in the circumferential direction. It’s worth mentioning that LAScd/LAScdc and LASct/LASctc are negative values since the atrial muscle fibres shorten during ventricular diastole and only the absolute value reflects the function condition. The three components of LA function can also be indicated by volumetric parameters. LAEF, representing the LA reservoir function, can be further divided into passive LAEF (pLAEF) and active LAEF (aLAEF) based on atrial contraction, reflecting conduit function and pump function respectively.

$$\mathrm{pLAEF}=\left(\mathrm{LAVmax}-\mathrm{LAVpreA}\right)/\mathrm{LAVmax}$$

$$\mathrm{aLAEF}=\left(\mathrm{LAVpreA}-\mathrm{LAVmin}\right)/\mathrm{LAVpreA}$$

For patients under AF rhythm, the LA lost active contraction and thus only pLAEF existed and was equalled to LAEF, which was also the case for LAScd/LAScdc.

### Statistical analysis

Continuous variables are expressed as mean ± standard deviation (SD) or median (quartiles) depending on the results of normality test. Kruskal-Wallis H test or one way ANOVA was used to assess difference among 3 groups when appropriate, followed by post hoc test with adjusted *p* value for multiple comparisons. Differences between two groups were compared using t test or wilcoxon rank-sum test. Categorical variables are presented as amount (percentage) and compared using Chi-square test or Fisher’s exact test where appropriate.

We randomly selected 30 3D volume datasets from control group, 60 from PAF group, 30 from Per-AF group and had the same operator as well as a different observer repeat the offline analysis at least one month after the initial measurements to assess intraobserver and interobserver variability. The intraclass correlation coefficient (ICC) were used to assess reproducibility.

Multi-linear regression was performed to determine whether LA function parameters were independently corelated with the presence and type of AF. Variables found significant in univariant analysis (*p* < 0.1) or considered clinically relevant were adjusted including age, body mass index (BMI), heart rate (HR), diastolic blood pressure (DBP), LVEF, E/e’, LV diastolic function grade, comorbidity, etc..

Correlation between two variables was analysed using Spearman rank correlation and the difference between 2 correlation coefficients were tested using z test.

Receiver operating characteristics (ROC) curves of different LA function parameters to identify PAF from non-AF subjects were constructed. Cutoff value was determined with the greatest Youden index (sensitivity + specificity − 1) and we calculated the positive predictive value (PPV) as well as negative predictive value (NPV) of different LA parameters in populations with PAF prevalence of 1 and 20%.

SPSS 22.0.0.0 (IBM, Armonk, New York, USA) was used for statistical analysis. *P* < 0.05 were considered statistically significant.

## Results

### Feasibility of 3D STE

Three hundred thirty four subjects including 59 non-AF subjects, 144 PAF and 131 Per-AF patients were initially enrolled in our study and completed conventional transthoracic echocardiographic exam as well as 3D STE. Five PAF as well as 22 Per-AF were then excluded during offline analysis process due to poor image quality, existence of artifacts or tachycardia leading to unsatisfying endocardial tracking. Eventually, 59 non-AF subjects, 139 PAF and 109 Per-AF patients were included in further analysis. The overall success rate of 3D STE was 91.9%.

### Description of study population

Baseline characteristics of the 3 groups were summarized in Table 1. The disease course of patients with Per-AF was longer than that of patients with PAF. There was no difference in terms of gender, height, weight, BMI, body surface area (BSA) or blood pressure at examination among three groups. However, subjects with Per-AF were older and had higher heart rate than the other two groups. Both AF groups had higher prevalence of hypertension, coronary artery disease (CAD) and stroke compared with control group meanwhile more subjects in Per-AF group were comorbid with congestive heart failure (CHF) and diabetic mellitus (T2DM). In addition, patients in PAF group had relatively lower CHA_2_DS_2_-VASc score, lower concentration of N terminal pro B type natriuretic peptide (NT pro-BNP), higher percentage of using type I and type III antiarrhythmic medication as well as ablation history compared to Per-AF group.

**Table 1:** Baseline characteristics among three groups

Variables	Control	PAF	Per-AF	*p*
	*n* = 59	*n* = 139	*n* = 109	
Age	60 (60,69)	68 (61,73)	69 (64,75)*	0.001
Male	36 (61%)	87 (63%)	73 (67%)	0.576
Height(cm)	164 (160,172)	167 (160,173)	168 (160,174)	0.448
Weight(kg)	66 ± 10	68 ± 11	69 ± 11	0.212
BMI(kg/m^2^)	24.1 ± 3.0	24.6 ± 3.1	24.8 ± 3.4	0.378
BSA(m^2^)	1.73 ± 0.16	1.76 ± 0.17	1.77 ± 0.19	0.263
SBP(mmHg)	130 (120,139)	133 (122,143)	132 (120,142)	0.105
DBP(mmHg)	81 (75,86)	80 (74,85)	80 (75,89)	0.583
HR(bpm)	69 (63,76)	67 (60,73)	77 (64,91)*†	< 0.001
Comorbidity				
CHF	0	4 (2.9%)	17 (15.6%)*†	< 0.001
Hypertension	13 (22%)	84 (60%)*	76 (70%)*	< 0.001
T2DM	4 (7%)	22 (16%)	30 (27%)*	0.002
CAD	0	24 (17%)*	36 (33%)*†	< 0.001
Stroke	0	17 (12%)*	34 (31%)*†	< 0.001
PVD	0	2 (1%)	4 (2%)	0.411
AF duration (month)	/	24 (2,60)	48 (5,91)	0.016
CHA_2_DS_2_-VASc	/	2 (1,4)	3 (2,5)	< 0.001
NT pro-BNP	/	120 (39,444)	956 (520,1918)	< 0.001
Antiarrhythmic medication				
Type I	/	32 (23%)	4 (4%)	< 0.001
Type II	/	52 (37%)	43 (39%)	0.743
Type III	/	35 (25%)	7 (6%)	< 0.001
Anticoagulant therapy	/	66 (47%)	46 (42%)	0.407
Ablation	/	17 (13%)	5 (5%)	0.029

*BMI* Body mass index, *BSA* Body surface area, *SBP* Systolic blood pressure, *DBP* Diastolic blood pressure, *HR* Heart rate, *CHF* Congestive heart failure, *T2DM* Type 2 diabetic mellitus, *CAD* Coronary artery disease, *PVD* Peripheral vascular disease, *NT pro-BNP* N terminal pro B type natriuretic peptide

*p after Bonferroni’s correction < 0.05 versus control group

†*p* after Bonferroni’s correction < 0.05 versus PAF group

### Conventional TTE derived LV and LA parameters

Results of conventional 2D echocardiography exam were shown in Table 2. The LV systolic function of subjects in Per-AF group were relatively compromised demonstrated by lower LVEF than subjects in the other two groups. The LV diastolic function were sequentially decreased among 3 groups. All 3 LA diameters significantly increased along with the progression of AF.

**Table 2:** Conventional TTE results among three groups

Parameters	Control	PAF	Per-AF	*p*
	*n* = 59	*n* = 139	*n* = 109	
LVEDD(mm)	48 (45,52)	49 (46,52)	48 (45,52)	0.608
LVEDV(ml)	64 (55,82)	72 (57,84)	65 (52,80)	0.118
LVESV(ml)	22 (18,30)	27 (20,32)	29 (22,36)*	0.001
LVEF(%)	64 (60,67)	62 (58,67)	54 (48,61)*†	<0.001
E(cm/sec)	66 (57,76)	70 (58,83)	95 (83,107)*†	<0.001
A(cm/sec)	86 (75,98)	78 (65,93)*	/	0.007
E/A	0.77 (0.69,0.86)	0.88 (0.70,1.12)*	/	0.002
Septal e’(cm/sec)	6 (6,8)	6 (5,8)	9 (7,10)*†	<0.001
Lateral e’(cm/sec)	9 (7,11)	9 (7,10)	12 (10,14)*†	<0.001
Septal a’(cm/sec)	9 (9,10)	9 (8,10)	/	0.264
Lateral a’(cm/sec)	11 (10,14)	11 (8,12)*	/	0.011
E/e’	8.3 (7.1,9.6)	9.3 (7.5,11.0)*	9.4 (7.1,11.4)*	0.026
TRMax(m/s)	2.31 (2.12,2.57)	2.47 (2.27,2.76)	2.48 (2.27,2.78)*	0.038
LVDF grade		*	*†	<0.001
Normal	56 (95%)	104 (75%)	58 (56%)	
Indeterminate	2 (3%)	20 (14%)	/	
Diastolic dysfunction	1 (2%)	15 (11%)	45 (74%)	
AP-LAD(mm)	35 (33,38)	39 (36,41)*	45 (42,47)*†	<0.001
SI-LAD(mm)	47 (44,52)	51 (47,55)*	60 (54,66)*†	<0.001
ML-LAD(mm)	34 (31,39)	38 (34,41)*	43 (39,47)*†	<0.001
2DLAVImax (ml/m^2^)	22 (18,26)	27 (21,31)*	42 (31,51)*†	<0.001

*LVEDD* Left ventricular end-diastolic diameter, *LVEDV* Left ventricular end-diastolic volume, *LVESV* Left ventricular end-systolic volume, *LVEF* Left ventricular ejection fraction measured by Biplane Simpson method, *E* Early filling velocity of trans-mitral Doppler flow, *A* Late filling velocity of trans-mitral Doppler flow, *E/A* E to A ratio, *Septal e’* Peak early diastolic mitral annular velocity at septal wall, *Lateral e’* Peak early diastolic mitral annular velocity at lateral wall, *Septal a’* Peak late diastolic mitral annular velocity at septal wall, *Lateral a’* Peak late diastolic mitral annular velocity at lateral wall, *E/e’* E to e’ ratio (e’ being the average value of septal e’ and lateral e’), *TRMax* Maximal tricuspid regurgitation velocity, *LVDF grade* Left ventricular diastolic function grade, *AP-LAD* Left atrial anteroposterior diameter, *SI-LAD* Left atrial suprainferior diameter, *ML-LAD* Left atrial mediolateral diameter, *2DLAVImax* LA maximal volume measured by Biplane Simpson method index to BSA

### Reproducibility of 3D STE derived parameters under sinus and AF rhythm

Ninety 3D volume data sets acquired under sinus rhythm and 30 under AF rhythm were randomly selected for reproducibility test. ICC as well as its 95% confidence interval (95%CI) for intraobserver and interobserver variability were summarized in Table 3. For data sets acquired under sinus rhythm, all 3D STE derived parameters except LAScd and LAScdc had excellent intraobserver and interobserver reproducibility demonstrated by an ICC greater than 0.75. On the other hand, 3D STE derived LA function parameters acquired under AF rhythm had only modest interobserver reproducibility in despite of great intraobserver reproducibility.

**Table 3:** Intraclass correlation coefficient for 3D STE derived parameters testing intraobserver and interobserver reproducibility under sinus or AF rhythm

ICC	Interobserver variability		Intraobserver variability	
Parameters	Sinus rhythm	AF rhythm	Sinus rhythm	AF rhythm
LAVmin(ml)	0.950 (0.926 ~ 0.967)	0.963 (0.921 ~ 0.982)	0.994 (0.991 ~ 0.996)	0.993 (0.986 ~ 0.997)
LAVmax(ml)	0.964 (0.943 ~ 0.977)	0.961 (0.919 ~ 0.981)	0.993 (0.989 ~ 0.995)	0.997 (0.994 ~ 0.999)
LAVpreA(ml)	0.945 (0.918 ~ 0.964)	/	0.978 (0.966 ~ 0.985)	/
LAVImax(ml/m^2^)	0.962 (0.940 ~ 0.976)	0.966 (0.929 ~ 0.984)	0.993 (0.989 ~ 0.995)	0.998 (0.995 ~ 0.999)
LAEV(ml)	0.850 (0.781 ~ 0.899)	0.799 (0.620 ~ 0.899)	0.967 (0.950 ~ 0.978)	0.903 (0.804 ~ 0.954)
LAEF(%)	0.863 (0.799 ~ 0.908)	0.657 (0.392 ~ 0.821)	0.968 (0.951 ~ 0.979)	0.855 (0.769 ~ 0.945)
LASr(%)	0.818 (0.733 ~ 0.877)	0.596 (0.305 ~ 0.785)	0.877 (0.818 ~ 0.918)	0.852 (0.708 ~ 0.929)
LAScd(%)	0.664 (0.531 ~ 0.766)	0.596 (0.305 ~ 0.785)	0.643 (0.502 ~ 0.750)	0.852 (0.708 ~ 0.929)
LASct(%)	0.753 (0.647 ~ 0.831)	/	0.861 (0.796 ~ 0.907)	/
LASrc(%)	0.850 (0.780 ~ 0.972)	0.705 (0.464 ~ 0.848)	0.950 (0.925 ~ 0.967)	0.908 (0.811 ~ 0.956)
LAScdc(%)	0.639 (0.498 ~ 0.747)	0.705 (0.464 ~ 0.848)	0.710 (0.589 ~ 0.800)	0.908 (0.811 ~ 0.956)
LASctc(%)	0.814 (0.730 ~ 0.874)	/	0.915 (0.873 ~ 0.971)	/

*LAVmin* Minimal LA volume, *LAVmax* Maximal LA volume, *LAVpreA* LA volume at the onset of atrial contraction, *LAVImax* Maximal LA volume index to BSA, *LAEV* LA emptying volume, *LAEF* LA emptying fraction, *pLAEF* Passive LA emptying fraction, *aLAEF* Active LA emptying fraction, *LASr* Peak atrial longitudinal strain, *LAScd* Early diastolic longitudinal strain, *LASct* Late diastolic longitudinal strain, *LASrc* Peak atrial circumferential strain, *LAScdc* Early diastolic circumferential strain, *LASctc* Late diastolic circumferential strain

All *p* value < 0.001

### Association between AF and LA function demonstrated by 3D STE derived parameters

#### LA structural and functional changes in subjects with PAF and per-AF

Comparison of 3D STE derived LA parameters among 3 groups were shown in Table 4. Note that LAVpreA, pLAEF, aLAEF, LASct and LASctc weren’t available in subjects under AF rhythm due to loss of atrial contraction. All four LA volume parameters were increased in PAF group compared with control group, and further increased in Per-AF group. LAEF, LASr and LASrc representing LA reservoir function decreased sequentially within the three groups. However, pLAEF reflecting LA conduit function seemed paradoxically higher in Per-AF group than in PAF group though no significant difference were shown by LAScd or LAScdc. LA pump function was notably impaired in patients with PAF even under sinus rhythm indicated by reduced aLAEF, LASct and LASctc.

**Table 4:** Changes of 3D STE derived LA function parameters in PAF and Per-AF

Parameters	Control	PAF	Per-AF	*p*
	*n* = 59	*n* = 139	*n* = 109	
LAVmin(ml)	19 (16,24.5)	30 (24,36)*	62 (46,77)*†	<0.001
LAVmax(ml)	41 (35,50)	52 (44,63)*	78 (62,98)*†	<0.001
LAVpreA(ml)	33 (26,38)	41 (36,50)*	/	<0.001
LAVImax(ml/m^2^)	24.9 (19.8,28.9)	29.5 (24.9,35.9)*	43.9 (34.9,54.8)*†	<0.001
LAEV(ml)	21 (17,26)	21 (16,27)	18 (13,21)*†	<0.001
LAEF(%)	53 (48,58)	42 (35,48)*	22 (18,25)*†	<0.001
pLAEF(%)	23 (19,28)	20 (14,24)*	22 (18,25)†	0.003
aLAEF(%)	39 (34,44)	27 (20,36)*	/	<0.001
LASr(%)	21 (17,26)	16 (12,20)*	7 (6,9)*†	<0.001
LAScd(%)	−10(−12,−7)	−8(−10,−6)*	−7(−9,−6)*	<0.001
LASct(%)	−11(−14,−9)	− 7(− 10,−4)*	/	<0.001
LASrc(%)	31 (26,36)	18 (13,24)*	8 (6,10)*†	<0.001
LAScdc(%)	−11(− 14,−6)	− 7(− 10,−5)*	−8(− 10,−6)*	<0.001
LASctc(%)	−20(−24,−15)	− 11(− 17,−7)*	/	<0.001

*p after Bonferroni’s correction < 0.05 versus control group

†*p* after Bonferroni’s correction < 0.05 versus PAF group

#### AF and higher burden of AF were independently associated with impairment of LA reservoir and pump function

Since there were some baseline imbalances among 3 groups and LA function were largely influenced by LV function, multivariant analysis was performed to illustrate LA morpho-functional changes independently associated with AF as well as AF type, and the partial regression coefficient(β) and adjusted *p* value were summarized in Table 5. It’s notable that pLAEF and LAScd no longer showed correlation with AF except that LAScdc was reduced in PAF group. Other than that, LA enlargement and impairment of the reservoir and pump function were independently associated with AF, and the remodeling was more significant in Per-AF group.

**Table 5:** Multi-linear regression of AF type and 3D STE derived LA parameters

Parameters	PAF(*n* = 139)		Per-AF(*n* = 109)	
	Partial regression Coefficient(β)	Adjusted p	Partial regression Coefficient(β)	Adjusted *p*
LAVmin(ml)	9.15 (3.33 ~ 14.97)	0.002	37.50 (29.57 ~ 45.43)	<0.001
LAVmax(ml)	6.81(−0.005 ~ 13.63)	0.050	32.43 (22.78 ~ 42.08)	<0.001
LAVpreA(ml)	3.63 (0.39 ~ 6.86)	0.028	/	/
LAVImax(ml/m^2^)	4.05 (0.01 ~ 8.10)	0.050	19.85 (14.04 ~ 25.66)	<0.001
LAEV(ml)	−0.93 (−2.84 ~ 0.98)	0.337	−1.72 (−4.08 ~ 0.65)	0.154
LAEF(%)	−9.49 (−11.91 ~ −7.06)	<0.001	−25.99 (− 29.59 ~ − 22.39)	<0.001
pLAEF(%)	− 1.79 (−3.71 ~ 0.12)	0.066	1.95 (− 0.90 ~ 4.80)	0.179
aLAEF(%)	−8.43(− 11.51 ~ −5.35)	<0.001	/	/
LASr(%)	−4.33 (−5.88 ~ −2.80)	<0.001	−11.46 (− 13.71 ~ − 9.21)	<0.001
LAScd(%)	0.57 (−0.53 ~ 1.67)	0.307	0.13 (−1.25 ~ 1.52)	0.852
LASct(%)	2.45 (1.03 ~ 3.87)	0.001	/	/
LASrc(%)	−10.68 (−12.83 ~ −8.54)	<0.001	− 18.90 (−22.08 ~ − 15.72)	<0.001
LAScdc(%)	2.27 (1.02 ~ 3.53)	<0.001	0.38 (−1.21 ~ 1.97)	0.635
LASctc(%)	6.91 (4.45 ~ 9.38)	<0.001	/	/

adjusted *p*: *p* value after adjusted for age, BMI, HR, DBP, LVEF, E/e’, LV diastolic function grade, presence of CHF, hypertension, T2DM, CAD, stroke, use of antiarrhythmic medication, etc.

#### 3D STE detected LA function impairments in subjects without LA enlargement

Fifty-five(93.2%) control, 113(74.8%) PAF and 38(25.2%) Per-AF in our study had normal LA sizes defined as LAVImax≤34 ml/m^2^ measured by 2D Biplane Simpson method according to the 2015 ASE guideline. We further analyzed the 3D STE derived LA parameters in this sub-group and results were shown in Table 6. Significant increases in LAVmin, LAVmax and LAVImax were observed among 3 groups along with the progression of AF. LAEF, LASr, LASrc were reduced in PAF group compared with the control group, and the reductions were more pronounced in Per-AF group. LAScd and LAScdc were decreased in both PAF and Per-AF groups. LA pump function was also significantly declined demonstrated by lower aLAEF and smaller LASct/LASctc in PAF subjects than control subjects.

**Table 6:** 3D STE derived LA function parameters in subjects with normal LA size (defined as 2DLAVImax ≤34 ml/m^2^)

Parameters	Control	PAF	Per-AF	*p*
	*n* = 55	*n* = 113	*n* = 37	
LAVmin(ml)	19 (15,24)	28 (22,34)*	45 (40,48)*†	<0.001
LAVmax(ml)	41 (34,49)	49 (43,57)*	61 (54,66)*†	<0.001
LAVpreA(ml)	31 (26,37)	39 (34,46)*	/	<0.001
LAVImax(ml/m^2^)	24.7 (19.7,27.8)	28.1 (23.7,31.5)*	31.6 (27.2,36.1)*†	<0.001
LAEV(ml)	21 (17,25)	21 (16,26)	16 (11,18)*†	<0.001
LAEF(%)	54 (48,58)	43 (37,49)*	24 (19,29)*†	<0.001
pLAEF(%)	24 (20,28)	20 (15,24)*	24 (19,29)†	<0.001
aLAEF(%)	40 (35,44)	27 (21,36)*	/	<0.001
LASr(%)	21 (18,26)	16 (13,20)*	8 (6,11)*†	<0.001
LAScd(%)	−10(−13,−7)	−8(−10,−6)*	−8(−11,−6)*	0.007
LASct(%)	−11(−14,−9)	−7(− 10,−4)*	/	<0.001
LASrc(%)	31 (26,36)	19 (14,25)*	8 (6,11)*†	<0.001
LAScdc(%)	−11(− 14,−7)	−7(− 11,−5)*	−8(− 11,−6)*	<0.001
LASctc(%)	−20(−24,−15)	−12(−18,−7)*	/	<0.001

*p after Bonferroni’s correction < 0.05 versus control group

†*p* after Bonferroni’s correction < 0.05 versus PAF group

### Notable correlations between volumetric function parameters and strain parameters representing the same LA function

As the three components of LA function could be derived from volumetric parameters or strain, we analyzed the relationship between LA emptying fraction and LA strain reflecting the same function and the correlation coefficients were shown in Table 7. It was notable that the correlation between LASrc and LAEF was significantly stronger than that between LASr and LAEF. Similarly, LAScdc and LASctc also correlated better with pLAEF and aLAEF respectively, compared with LAScd and LASct.

**Table 7:** Correlations between LA volumetric function parameters and strains indicating the same function

LA function	Parameters	r with	r with	r with	*p*
		LAEF (*n* = 307)	pLAEF (*n* = 198)	aLAEF (*n* = 198)	
Reservoir	LASr	0.891*	/	/	<0.001
	LASrc	0.947*			
Conduit	LAScd		−0.671†	/	<0.001
	LAScdc		−0.884†		
Pump	LASct			−0.768✦	<0.001
	LASctc			−0.914✦	

*r* Correlation coefficient

Correlation coefficients marked with the same symbols (*/†/✦) suggest significant difference between their comparison

### 3D STE derived parameters can be used to differentiate subjects with PAF from non-AF subjects

We further sought to assess whether 3D STE derived function parameters could be used to differentiate PAF patients from subjects with no recorded history of AF. ROC curves were constructed and related parameters were demonstrated in Table 8. LASrc yielded the largest AUC for identification of PAF, with 75.5% sensitivity and 81.4% specificity when the cutoff value was set at 24%. PPV and NPV of the parameters with an AUC over 0.8 when applied to populations with AF prevalence of 1% or 20% were calculated and summarized in Table 9.

**Table 8:** ROC analysis for differentiating PAF patients from non-AF subjects with 3D STE derived parameters

Parameters	AUC(95%CI)	Cutoff value	Sensitivity(95%CI)	Specificity(95%CI)
LAVImax(ml/m^2^)	0.703 (0.627 ~ 0.780)	27.8	61.9 (53.3 ~ 70.0)	72.9 (59.7 ~ 83.6)
LAVmin(ml)	0.823 (0.762 ~ 0.884)	21	86.3 (79.5 ~ 91.6)	67.8 (54.4 ~ 79.4)
LAEF(%)	0.830 (0.770 ~ 0.891)	46.5	69.8 (61.4 ~ 77.3)	84.7 (73.0 ~ 92.8)
pLAEF(%)	0.639 (0.551 ~ 0.726)	22.3	69.1 (60.7 ~ 76.6)	57.6 (44.1 ~ 70.4)
aLAEF(%)	0.826 (0.768 ~ 0.884)	33.7	69.1 (60.7 ~ 76.6)	79.7 (67.2 ~ 89.0)
LASr(%)	0.758 (0.685 ~ 0.830)	18	69.1 (60.7 ~ 76.6)	72.9 (59.7 ~ 83.6)
LASrc(%)	0.850 (0.793 ~ 0.908)	24	75.5 (67.5 ~ 82.4)	81.4 (69.1 ~ 90.3)
LAScd(%)	0.635 (0.549 ~ 0.722)	−11	81.3 (73.8 ~ 87.4)	42.4 (29.6 ~ 55.9)
LAScdc(%)	0.687 (0.602 ~ 0.772)	−9	65.5 (56.9 ~ 73.3)	69.5 (56.1 ~ 80.8)
LASct(%)	0.741 (0.669 ~ 0.814)	−8.5	59.7 (51.1 ~ 67.9)	79.7 (67.2 ~ 89.0)
LASctc(%)	0.828 (0.768 ~ 0.887)	−14	62.6 (54.0 ~ 70.6)	86.4 (75.0 ~ 94.0)

*AUC* Area under the curve, *95%CI* 95% confidence interval

All *p* < 0.001

**Table 9:** PPV and NPV of LA parameters when applied to populations with AF prevalence of 1% or 20%

Parameters	Prevalence 1%		Prevalence 20%	
	PPV(95%CI)	NPV(95%CI)	PPV(95%CI)	NPV(95%CI)
LAVImax(ml/m^2^)	2.3 (1.5 ~ 3.4)	99.5 (99.3 ~ 99.6)	36.3 (29.7 ~ 43.6)	88.4 (85.8 ~ 90.6)
LAVmin(ml)	2.6 (1.8 ~ 3.8)	99.8 (99.7 ~ 99.9)	40.1 (34.3 ~ 46.2)	95.2 (92.8 ~ 96.8)
LAEF(%)	4.4 (2.4 ~ 7.8)	99.6 (99.5 ~ 99.7)	53.3 (43.2 ~ 63.1)	91.8 (89.6 ~ 93.6)
aLAEF(%)	3.3 (2.0 ~ 5.4)	99.6 (99.5 ~ 99.7)	46.0 (37.5 ~ 54.6)	91.2 (88.8 ~ 93.1)
LASrc(%)	3.9 (2.3 ~ 6.6)	99.7 (99.6 ~ 99.8)	50.4 (41.4 ~ 59.3)	93.0 (90.8 ~ 94.7)
LAStc(%)	4.5 (2.4 ~ 8.3)	99.6 (99.4 ~ 99.7)	53.5 (42.6 ~ 64.1)	90.2 (88.1 ~ 92.0)

*PPV* Positive predictive value, *NPV* Negative predictive value

## Discussion

In this study, we aimed to comprehensively assess LA morphofunctional remodeling using 3D STE which was shown to be feasible and reproducible. Our main finding is that AF is independently associated with LA enlargement as well as impairment of LA reservoir and pump function but not conduit function. Besides, 3D STE derived function parameters, especially LAVmin, LAEF, aLAEF, LASrc and LASctc can be used to differentiate PAF from non-AF subjects with good sensitivity and specificity.

### Feasibility of 3D STE in assessing LA function

In our study, the success rate of 3D STE analysis in each groups was 100, 96 and 83% respectively. In terms of reproducibility, both intraobserver and interobserver reproducibility were excellent when conducted under sinus rhythm, however, in patients with Per-AF, the interobserver reproducibility was less satisfying. The reasons were as follows: 1) LA enlargement was more significant in Per-AF and thus wider sector angle was needed during acquisition to include the entire LA which lowered the frame rates and image quality, 2) multi-beat acquisition might be feasible under AF rhythm, but the reconstruction effect would be largely compromised, 3) only single-beat acquisition can be used with obvious heart rate variation which significantly lowered the frame rates, 4) patients with Per-AF tended to have higher heart rates as shown by our study, and thus higher frame rates were needed in order to improve temporal resolution which again compromised image quality. To sum up, practically it’s more difficult to obtain 3D images with good quality in subjects with Per-AF, which lowers the accuracy of automatic LA contour recognition and tracing by the software, and therefore more manual correction is needed which is not always easy and introduces more error in measurements since there still lack widely accepted standard operational guide of this new technique, especially in subjects with Per-AF.

Echopac 4D Auto LAQ by GE Health, as a 3D STE software package dedicated to LA, was not available until recent years. Experience in using this software is scarce. One study demonstrated LA functional changes in end stage renal dysfunction in a total of 71 subjects, but didn’t discuss the usage of the software at the same time [25]. To our knowledge, our study is the first to analyze the feasibility and reproducibility of this software as well as its utility to assess LA morpho-functional remodeling in patients with both PAF and Per-AF, at the meantime with a decent sample size and thus filled certain gap in this field.

### LA structural and functional changes associated with AF and AF pattern

As LA function is affected by many factors such as age, gender, BMI, comorbidity, LV function status, etc., therefore, in order to demonstrate LA remodeling independently associated with AF, confounders were adjusted based on univariant linear regression and clinical judgement. Our study showed that impairment of LA reservoir and contraction function was independently associated with PAF, additionally, LA reservoir function was further decreased in Per-AF patients. However, pLAEF and LAScd were not independently associated with PAF or Per-AF, suggesting that AF has relatively small direct impact on LA conduit function. Interestingly, we noticed that pLAEF was even higher in Per-AF group compared to PAF group, which we believe was caused by different calculating methods of pLAEF between 2 groups as it was considered equal to LAEF under AF rhythm. Therefore, comparison of LA conduit function between subjects under different rhythm is not suitable.

Our results are consistent with previous studies, demonstrating association between AF and enlarged LA volumes as well as impaired LA function indicated by conventional 2D echocardiography derived volume measurements [10, 26] or longitudinal LA strain and strain rate derived from 2D STE [27] or DTI [28]. However, 3D STE, as a novel technique combining real-time echocardiography with speckle tracking analysis, is a lot less applied in LA function assessment compared to LV. Mochizuki et al. used 2D STE and 3D STE to compare atrial deformation and synchrony in healthy subjects and patients with AF and found that 3D STE was less time consuming and more sensitive [16]. Significant decreases in longitudinal, circumferential and area strain across AF types were detected, however, confounders were not adjusted in their study in despite of some significant imbalances at baseline and thus couldn’t yet manifest AF-induced changes in LA strain as they tried to address in their article. The results of our study, with a much greater sample size, is a valuable supplement to theirs and more conclusive as explicating LA structural and functional changes independently associated with AF. A sub-study of the MYGYAR-path study conducted by Chadaide et al. compared 3D STE derived LA strain parameters in 20 AF patients and 11 healthy controls. Decrease of peak circumferential and radial strain was shown in all LA segments while reduction of peak longitudinal strain was only detected in mid and superior LA segments, and no difference was found in terms of LAEF [29]. However, their results may hold certain bias due to small sample size. Furukawa et al. sought to assess the effect of PAF on top of hypertension on LA function using 3D STE (44 PAF + HTN vs. 50 non-AF HTN), and demonstrated decreased peak atrial longitudinal strain, LAEF and synchrony compared to hypertensive patients without PAF which indicated diminished LA compliance and electro-mechanical abnormality [30]. The above studies shared one common limitation that they used 3D STE software originally designed for LV which was prone to introducing errors. Good news is that GE Health has recently launched 3D STE software package dedicated to LA, and to our knowledge, our study is the first to explore its application in AF population including subjects with Per-AF whom were usually excluded in many studies, and thus provides valuable experience.

### Relationship between LA volumetric function parameters and strain

Echopac 4D Auto LAQ allows simultaneous analysis of LA volume and strain, and therefore we were able to compare the relationship between different LA function parameters. The three components of LA function can be either indicated by volumetric function parameters (LAEF, pLAEF, and aLAEF) or strain parameters (LASr/LASrc, LAScd/LAScdc, and LASct/LASctc). It’s not surprising to find strong correlation between LA emptying fraction and its corresponding strain parameter that represents the same LA function, however, on top of that, we found that the correlation between LA emptying fraction and its corresponding circumferential strain was always significantly stronger than that with the longitudinal strain, which we believe suggest that changes of LA deformation in the circumferential direction might have greater impact on LA global hemodynamic function. To our knowledge, our study is the first to compare the relationship between LA volumetric function parameters and longitudinal strain with the relationship between LA volumetric function parameters and circumferential strain since few studies were able to provide all these parameters at the same time using other techniques. At present, many studies are focusing on 2D STE derived LA longitudinal strain and its prognostic value for various adverse cardiovascular outcome, however, we assume that LA circumferential strain might possibly hold greater value, and therefore we call for more studies with this index in the future.

### AF related fibrosis and LA remodeling

LA enlargement and fibrosis are hallmark of AF which not only initiate but also contribute to the maintenance and progression of AF [4, 31]. The degree of LA fibrosis can be evaluated via delayed-enhancement cardiac magnetic resonance imaging (DE-CMR), however its use in daily clinical practice is limited due to its high cost, operational complexity and significant time consumption [32]. Till now, echocardiography remains the most useful and convenient tool to evaluate cardiac structure and function. Since it’s now believed that fibrosis is associated with LA remodeling and decrease of LA compliance, LA function parameters assessed by echocardiography can be considered as a surrogate for LA fibrosis. There have been some studies supporting this idea. Cameli et al. found that peak atrial longitudinal strain (PALS) derived from 2D STE had strong inverse relationship with the extent of LA fibrosis (r = − 0.82) based on histological sample obtained from valve replacement surgery in 46 AF patients with severe mitral regurgitation while LAVImax and LAEF showed medium association with LA fibrosis (r = 0.51 and − 0.61, respectively) [33]. Another study by Kuppahally et al. demonstrated that strain value of LA mid-lateral wall was inversely related to the extent of LA fibrosis detected by DE-CMR as well as AF burden [34]. Therefore, comprehensive assessment of LA function via 3D STE can provide doctors with more disease information of different AF patients.

### Identification of PAF using LA function parameters

Another important finding of our study is that LA volume and function parameters can be used to distinguish PAF from non-AF subjects with great accuracy, sensitivity and specificity.

According to current guideline, diagnosis of AF should be made based on a standard 12-lead ECG or a single-lead ECG [35]. The current prevalence of AF is estimated to be 2–4% [1], however, since some AF episodes are completely asymptomatic, or instantly self-terminate before ECG examinations are conducted, many AF patients are undetected. AF can lead to some fatal and highly-disabling complications such as stroke and congestive heart failure, which can be prevented or managed through medication. Therefore, it’s of great importance to identify those PAF patients who visited the clinics under sinus rhythm and make timely diagnosis. In the REVEAL AF study, insertable cardiac monitor (ICM) was used to screen for AF in patients with high risk for a mean observation period of 22.5 months. The detection rate of AF at 18 months was 29.3% which further increased to 40.0% if the monitoring period was prolonged to 30 months, revealing a surprising amount of undetected AF via conventional 12-lead ECG or regular 24 h ambulatory ECG monitoring [36]. The CRYSTAL-AF study aimed to detect underlying AF in patients with cryptogenic stroke using ICM and the detection rate at 6, 12, and 36 months was 8.9, 12.4 and 30%, respectively [37]. It’s easy to understand that the more intense the screening strategy is, the more undiagnosed AF can be detected, yet with higher cost. Therefore, the major issue is to determine the high risk population that truly needs prolonged rhythm monitoring. In our opinion, LA function parameters are useful under this scenario because unlike ECG abnormalities of PAF patients which is “paroxysmal”, changes of LA function are stable and can be detected by echocardiography under sinus rhythm, even before morphology changes take place, and thus can help to identify subjects with potentially underlying AF. In this study, we calculated the PPV and NPV of LA function parameters to identify PAF patients in populations with different AF prevalence. As for the general public with an estimated PAF prevalence of 1% [1, 38], the NPV of various LA volume/function parameters was excellent (> 99%), and therefore can be used to effectively rule out PAF and so we know who don’t need intensified ECG monitoring. On the other hand, in populations with a stroke history where the prevalence of AF is approximately 20% [39], the PPV of LAEF and LASrc for identification of PAF exceeded 50%, which is of great value because current commonly used tools for investigating cardiogenic stroke are echocardiography and 24 h ambulatory electrocardiogram with the purpose to find relevant structure abnormalities (such as patent foramen ovale, LA thrombus, endocardial vegetations, etc.) and AF. However, it’s highly likely that no AF episodes occur during monitoring period after the stroke event, which may lead to misclassification of the stroke type as cryptogenic stroke and delay the initiation of appropriate anticoagulant therapy. What we propose is that comprehensive assessment of LA volume and function using 3D STE can further suggest whether the patients still hold great chance (> 50%) of underlying AF even when Holter results are negative, and therefore require prolonged ECG monitoring. On the contrary, intensified AF screening can be reasonably saved when LA volume and function are within normal ranges. It needs to be point out that our aim is not to use LA function parameters derived from echocardiography to directly diagnose AF, but to help determine the optimal screening strategy for different individuals. In this way, valuable medical resources can be reasonably allocated.

### Advantages and limitations of 3D STE

STE is the current preferred method for strain measurement compared to TDI, as the latter is angle dependent, time-consuming due to requirement of wall-by-wall sampling, relatively more dependent on pre-load, and poor in reproducibility. Up till now, 2D STE is the most commonly used method to obtain strain, however, it faces problems such as lacking consensus on acquisition and offline analysis, foreshortening of the 2D images, the out-of-plane phenomenon, etc.. The Echopac 4D Auto LAQ we used in our study is a 3D STE software package dedicated to the LA, which technically overcomes the inherent disadvantages of 2D STE. Its high degree of automation saves the need to manually trace LA endocardium, which not only reduces analysis time, but also greatly improves reproducibility. In addition, the information it provides is more elaborate, including reliable LA phasic volumes which is not based on geometric assumption, LA volumetric function parameters as well as LA strain in multiple directions. On the other hand, 3D STE faces certain challenges as well. As it’s a rather new technique, it lacks operation consensus and experience. Besides it has a relatively higher requirement for image quality that might lead to unsuccessful analysis or unreliable results if not fulfilled. Specific software and transducer are needed to perform 3D STE and values vary between venders which limit its application in clinical practice. However, since 3D echocardiography is a promising tool in the future, and the operation of 3D STE is actually easier compared to 2D STE, yet providing a lot more information, we believe this technique hold great value in both clinical practice and scientific research.

### Limitations

Our study is a single centre, cross-sectional study took place in a public teaching hospital and though we tried our best to enrol patients with AF continuously during the study period, some might still be missed due to various reasons, and thus our study may carry certain bias. The classification of PAF and Per-AF was made based on patients’ description of their symptoms combining with their previous ECG and Holter reports, and therefore misclassification between groups might exist. We detected stronger correlations between LA circumferential strains and volumetric function parameters, but the underlying mechanism couldn’t yet be drawn from this study. LA circumferential strains might possibly hold greater value than longitudinal strains, but more studies will be needed in the future.

## Conclusions

3D STE allows us to comprehensively assess LA function with good feasibility and reproducibility, but more experience will be needed in the future especially in patients with Per-AF. Changes of LA deformation in the circumferential direction might have greater impact on LA global hemodynamic function. LA enlargement as well as impairment of LA reservoir and pump function are independently associated with AF, and are more significant with higher AF burden. LA volume and function parameters can be used to identify PAF with good accuracy and thus can guide AF screening strategy in clinical practice. As LA pump function is lost in patients under AF rhythm and conduit function is relatively less affected by AF, we believe parameters representing LA reservoir function, especially LASrc, hold the greatest clinical value. Therefore, we call for more studies to investigate the prognostic value of this index in the future.

## Abbreviations

2D STE: Two dimensional speckle tracking echocardiography
2DLAVImax: Left atrial maximal volume measured by Biplane Simpson method index to body surface area
2DLAVmax: Left atrial maximal volume measured by Biplane Simpson method
3D STE: Three dimensional speckle tracking echocardiography
A: Late filling velocity of trans-mitral Doppler flow
AF: Atrial fibrillation
aLAEF: Active left atrial emptying fraction
AP-LAD: Left atrial anteroposterior diameter
AUC: Area under the curve
BMI: Body mass index
BSA: Body surface area
CAD: Coronary artery disease
CHF: Congestive heart failure
CI: Confidence interval
DBP: Diastolic blood pressure
DE-CMR: Delayed-enhancement cardiac magnetic resonance imaging
E: Early filling velocity of trans-mitral Doppler flow
E/A: E to A ratio
E/e’: E to e’ ratio (e’ being the average value of septal e’ and lateral e’)
ECG: Electrocardiogram
ESC: European Society of Cardiology
HR: Heart rate
ICC: Intraclass correlation coefficient
ICM: Insertable cardiac monitor
LA: Left atrium
LAEF: Left atrial emptying fraction
LAEV: Left atrial emptying volume
LAScd: Longitudinal left atrial strain conduit
LAScdc: Circumferential left atrial strain conduit
LASct: Longitudinal left atrial strain contraction
LASctc: Circumferential left atrial strain contraction
LASr: Longitudinal left atrial strain reservoir
LASrc: Circumferential left atrial strain reservoir
Lateral a’: Peak late diastolic mitral annular velocity at lateral wall
Lateral e’: Peak early diastolic mitral annular velocity at lateral wall
LAVImax: Maximal left atrial volume index to body surface area
LAVmax: Maximal left atrial volume
LAVmin: Minimal left atrial volume
LAVpreA: Left atrial volume at the onset of atrial contraction
LV: Left ventricle
LVDF: Left ventricle diastolic function
LVEDD: Left ventricular end-diastolic diameter
LVEDV: Left ventricular end-diastolic volume
LVEF: Left ventricular ejection fraction
LVESV: Left ventricular end-systolic volume
ML-LAD: Left atrial mediolateral diameter
NPV: Negative predictive value
NT pro-BNP: N terminal pro B type natriuretic peptide
PAF: Paroxysmal atrial fibrillation
PALS: Peak atrial longitudinal strain
Per-AF: Persistent atrial fibrillation
pLAEF: Passive left atrial emptying fraction
PPV: Positive predictive value
PVD: Peripheral vascular disease
ROC: Receiver operating characteristics
SBP: Systolic blood pressure
SD: Standard deviation
Septal a’: Peak late diastolic mitral annular velocity at septal wall
Septal e’: Peak early diastolic mitral annular velocity at septal wall
SI-LAD: Left atrial suprainferior diameter
STE: Speckle tracking echocardiography
T2DM: Type 2 diabetic mellitus
TDI: Tissue doppler imaging
TRMax: Peak tricuspid regurgitant velocity
TTE: Transthoracic echocardiography

## Footnotes

1 The study was temporarily suspended from 2020.1.15 to 2020.5.31 due to COVID-19.
